# Glycine-driven abiotic humification: Differential mediation of montmorillonite and kaolinite on the structural evolution of humic-like substances

**DOI:** 10.1371/journal.pone.0359311

**Published:** 2026-09-30

**Authors:** Chunfeng Shi, Zihan Zheng, Haihang Sun

**Affiliations:** College of Agriculture, Jilin Agricultural Science and Technology College, Jilin, China; National Research and Innovation Agency, INDONESIA

## Abstract

Abiotic humification via the Maillard reaction represents an indispensable geochemical process driving humic-like substance (HLS) formation and potential soil organic carbon (C) stabilization. Clay minerals act as critical modulators of this process, yet the differential mediating mechanisms of montmorillonite (Mont) and kaolinite (Kao)—two dominant soil clay minerals with distinct crystal structures—on glycine (Gly)-driven structural evolution of HLS remain poorly understood. Specifically, how Gly dosage couples with clay mineral types to modulate condensation reactions and structural differentiation of HLS has not been systematically clarified. In this study, a sterile abiotic reaction system was established with glucose and catechol as co-precursors, and five Gly concentration gradients (0, 0.03, 0.06, 0.12, and 0.24 mol/L) were set to investigate the regulatory effects of Gly on Maillard-derived HLS formation and structural evolution under Mont and Kao mediation. A combination of ultraviolet-visible spectroscopy, total organic carbon (TOC) analysis, elemental analysis, Fourier transform infrared (FTIR) spectroscopy, and partial least squares structural equation modeling (PLS-SEM) was applied to characterize HLS aromatic condensation, mineral–organic interactions, and abiotic humification pathways. Results showed that elevated Gly concentrations continuously increased the supernatant E_4_/E_6_ ratio and TOC content; this trend reflected enhanced adsorption of highly aromatic HLS fractions onto mineral surfaces, leaving simpler-structured components in the aqueous phase. The E_4_/E_6_ *A*_*max*_ relative increment reached 261.3% in Kao system versus 148.3% in Mont system, while Mont maintained more stable kinetic inflection *x*_*0*_ values with weaker fluctuation across Gly gradients. The optimal Gly concentration for humic-like acid (HLA) C (C_HLA_) accumulationwas 0.06 mol/L in both clay systems, where the maximum C_HLA_ value of Kao group reached 3.00 g/kg, 69.5% higher than the 1.77 g/kg observed in Mont treatments. Over the full incubation period, the average C_HLA_/fulvic-like acid (FLA) C (C_FLA_) ratio of Kao system increased by 492.8%, merely 160.5% for Mont; Mont-derived HLA exhibited consistently higher H/C atomic ratios (1.28–1.94) than Kao HLA (0.94–1.83), verifying weaker aromatic condensation of Mont-produced humic fractions. Kao likely facilitated higher aromatic condensation, molecular maturity, and C_HLA_/C_FLA_ ratio of HLS, whereas Mont restricted macromolecular polymerization through interlayer spatial confinement, producing HLA with higher H/C ratios and simpler molecular structures. FTIR spectra confirmed divergent responses of the two clay minerals to Gly gradients and strengthened mineral–organic coupling at the optimal 0.06 mol/L Gly dosage, with Kao displaying a wider fluctuation range (0.50–5.99) of polysaccharide C–O band intensity than Mont (0.91–1.01). PLS-SEM further verified that Gly addition directly drove early HLS formation, and mineral–organic coupling may serve as a key intermediate pathway related to HLS structural evolution and mineral-associated C retention. These findings likely reveal the structure-dependent regulatory patterns of typical soil clay minerals in abiotic humification under laboratory simulation, providing a preliminary mechanistic reference for optimizing precursor conditions to promote soil C stabilization.

## 1. Introduction

Abiotic humification is an indispensable and pervasive geochemical process in terrestrial ecosystems, rather than merely a complementary pathway to biotic humification [1]. It dominates continuous humic-like substances (HLS) production under low-microbe harsh habitats, sustaining ecosystem stability and mediating global carbon (C) sequestration and nutrients biogeochemical cycling [2]. As the core end-product of abiotic humification, HLS exert critical ecological functions, such as soil C fixation, pollution remediation, and nutrient retention. Clarifying the formation and structural evolution of abiotic HLS is therefore critical to enrich humification theory and quantify their environmental ecological impacts. Clay minerals serve as core regulators of abiotic humification due to their large specific surface area, high cation exchange capacity, and inherent catalytic properties. Montmorillonite (Mont) and kaolinite (Kao) are the two most widespread soil clays; their discrepant crystal frameworks, surface physicochemical properties, and interfacial reactivity are predicted to generate contrasting humification regulatory effects [3]. The Maillard reaction between amino acids and reducing sugars is confirmed as the primary abiotic pathway for natural HLS formation [1]. Glycine (Gly), the simplest and most ubiquitously soil amino acid, functions as a nitrogen(N)-containing precursor that initiates and drives the Maillard reaction during abiotic humification [4]. Nevertheless, it remains poorly understood whether and how Gly dosage couples with different clay types to modulate the condensation reaction and structural differentiation of HLS. In particular, the differential mediation mechanisms of Mont and Kao on the evolution of the molecular structure of Gly-driven HLS are still unclear [5].

Recent advances in abiotic humification research have further confirmed the Maillard reaction as a core pathway for the formation of HLS, with an increasing focus on the synergistic effects of mineral catalysts and Maillard reaction precursors (MRPs). Mineral-mediated regulation of the Maillard reaction has emerged as a research hotspot, encompassing metal oxides, clay minerals, and synthetic catalysts [6]. Reactive iron (Fe) and manganese (Mn) oxides could accelerate the Maillard reaction by up to two orders of magnitude via geopolymerization, converting glucose (Glu) and Gly into recalcitrant aromatic compounds, thereby contributing significantly to global sedimentary C preservation [7]. In addition, metal oxides, such as CuMgFe layered double oxides, efficiently enhanced polyphenol-Maillard humification; the addition of Gly further promoted C–N and C–C bond formation, yielding HLS with higher aromaticity and richer O-containing functional groups [8]. Notably, existing mineral-catalyzed Maillard humification studies have predominantly focused on redox-active metal oxides [1,2,6], while investigations of layered silicate soil clay minerals remain relatively sparse, and systematic comparisons between Mont and Kao systems are still underexplored. Studies using gibbsite and zeolite cannot represent the two most abundant agricultural soil clays, and lacked long-term dynamic monitoring of HLS C pool partitioning and kinetic model fitting of humification progression [9]. To date, few studies have applied PLS-SEM to disentangle and quantify the Gly dosage → mineral-organic coupling → HLS structural evolution → soil C sequestration cascade pathway separately for Mont and Kao systems. Gly, as a ubiquitous amino donor in soils, exhibits distinct concentration-dependent regulatory effects on mineral-catalyzed humification: Li et al. [9] demonstrated in a gibbsite-mediated system that low Gly concentrations (0–0.06 mol/L) facilitated the polymerization of catechol (Cat) and Glu into humic-like acid (HLA), whereas moderate to high concentrations (0.12–0.24 mol/L) optimized aromatic condensation, evidenced by a reduced E_4_/E_6_ ratio, and enriched O-containing functional groups; excessive Gly acted as a N donor to lower the C/N ratio of HLA. Even in non-catalytic systems, Gly served as a key Maillard reaction precursor, participating in polyphenol-Maillard polycondensation under light-limited conditions and influencing HLS composition (e.g., enrichment of aliphatic moieties) [10]. Additionally, amino acid types and concentrations were confirmed to modulate humification pathways: aromatic amino acids promoted HLA accumulation in Maillard reaction systems, whereas lysine enhanced humic acid (HA) aromatization in phenolic-protein systems, underscoring the specificity of amino acid regulation [11]. Recent studies have further verified that the Maillard reaction dominates HLA formation during the thermophilic phase of aerobic fermentation (where microbial participation is minimized), confirming its pivotal role in abiotic humification [12].

Despite these advancements, there is still room for improvement in addressing some relevant challenges. First, most previous studies on mineral-mediated Maillard reactions have not fully focused on the regulatory role of single amino acid dosage gradients, which makes it somewhat difficult to clarify the quantitative effect of amino acid concentration on HLS formation under mineral mediation. Second, the differential mediating mechanisms of Mont and Kao in the Maillard reaction—particularly how they modulated the response of HLS aromatic condensation degree and structure to changes in Gly dosage—have not been fully elucidated yet. In view of these, this study established a sterile abiotic reaction system with Glu and Cat as co-precursors and set up five gradient concentrations of Gly (0, 0.03, 0.06, 0.12, and 0.24 mol/L) to explore the regulatory effects of Gly on the formation and structural evolution of Maillard reaction-derived HLS under the mediation of Mont and Kao. A combination of ultraviolet-visible (UV-Vis) spectroscopy, total organic carbon (TOC) analysis, elemental analysis, Fourier transform infrared (FTIR) spectroscopy, and partial least squares structural equation modeling (PLS-SEM) was adopted to characterize the aromatic condensation of HLS, mineral–organic interactions, and the abiotic pathway of HLS formation. This study aimed to clarify the differential mediation mechanisms of Mont and Kao in abiotic humification and the dose-dependent regulation of Gly, and to provide a molecular-level theoretical basis for understanding soil C sequestration and the ecological functions of HLS.

To systematically highlight the research progress and unique contributions of the current work compared with prior mineral-mediated abiotic humification reports, a comparative summary table is provided below (Table 1). Most existing studies focused on metal oxide catalysts or adopted single mineral systems without amino acid concentration gradients, and few works decoupled the independent regulatory effects of montmorillonite and kaolinite. This study innovatively establishes independent Mont and Kao incubation systems across five Gly concentration gradients, integrates dynamic kinetic fitting, multispectral characterization and PLS-SEM pathway quantification, and advances current mechanistic understanding of the crystal-structure-dependent differential mediation of typical soil clays on Gly-driven Maillard humification.

**Table 1:** Comparative summary of previous mineral-mediated Maillard/abiotic humification studies and innovations of the present research.

Reference	Catalyst/Mineral Type	Amino acid setting	Experimental gradient design	Detection indicators	Core research limitation	Innovations of this study
Li et al. 2025 [9]	Gibbsite (metal oxide hydroxide)	Glycine Single gradient (0–0.24 mol/L)	Only Single mineral system	E_4_/E_6_, Elemental ratio, FTIR	No comparison of Two typical soil clay minerals; lacking kinetic fitting and PLS-SEM pathway quantification	Compare Mont & Kao Two dominant clays; adopt Logistic5/Boltzmann kinetic modeling; construct PLS-SEM to clarify mineral-organic coupling cascade
Ashraf et al. 2025 [10]	No mineral catalyst	Glycine single concentration	Non-catalytic blank system only	HLS elemental composition	Ignore mineral interfacial regulation; no clay mineral contrast test	Focus on two typical layered silicate clays, quantify interlayer confinement vs surface catalysis effects
Chen et al. 2025 [6]	Fe/Mn metal oxides	Mixed amino acid precursors	No concentration gradient of single amino acid	Spectroscopy, aromaticity	Target metal oxides instead of soil clay minerals; cannot reflect agricultural soil humification process	Take glycine (universal soil amino acid) as single variable, set five concentration gradients to quantify dose-effect relationship
Fukuchi et al. 2010 [13]	Natural zeolite	Fixed glycine concentration	No Gly concentration gradient	Static FTIR spectrum	Lack dynamic incubation kinetic monitoring; no differentiation of expandable/non-expandable clay structures	Long-term dynamic sampling (0–360 h); distinguish expandable Mont (2:1) and non-expandable Kao (1:1) crystal structure effects
Miura et al. 2009 [14]	Mont, Kao mixed system	Single fixed Gly dosage	No Gly concentration gradient; static endpoint test	Only color darkening degree	Cannot decouple mineral type × Gly concentration interaction; no quantitative carbon pool partitioning	Separate Mont and Kao independent systems; quantify C_HLA_/C_FLA_ carbon pool allocation; compare kinetic parameters of two clays

## 2. Materials and methods

### 2.1. Preparation of materials

Mont and Kao were purchased from Sinopharm Chemical Reagent Co., Ltd. (Shanghai, China). Prior to use, the minerals were pretreated to remove impurities by grinding, sieving through a 0.01 mm mesh, and drying under vacuum at 60°C for 24 h to ensure consistent physicochemical properties. Gly (analytical grade), Cat (analytical grade), Glu (analytical grade), hydrogen peroxide (H_2_O_2_, analytical grade), phosphoric acid (H_3_PO_4_), and other reagents were also purchased from Sinopharm Chemical Reagent Co., Ltd. (Shanghai, China) and used without further purification. Preparation of 0.2 mol/L (pH 8.0) phosphate-buffered saline: Next, 5.3 mL of 0.2 mol/L (NaH_2_PO_4_) and 94.7 mL of 0.2 mol/L (Na_2_HPO_4_) were mixed thoroughly, and 0.02% thimerosal (C_9_H_9_HgNaO_2_S) was added.

### 2.2. Experimental design

To prioritize abiotic transformation as the dominant process, strict sterile conditions were maintained throughout the experiment. All glassware, phosphate-buffered saline, and other relevant experimental apparatus were subjected to autoclaving at 121°C for 30 min for sterilization. The experiment was carried out in 500-mL conical flasks with two mineral treatment groups (Kao and Mont), five Gly concentration gradients, and a dedicated CK for each mineral type.

Prior to the formal experiment, a pre-experiment with a wider Gly concentration range (0, 0.03, 0.06, 0.12, 0.24, 0.36, 0.48 mol/L) was conducted to screen the effective concentration interval. Results showed that C_HLA_ accumulation exhibited a unimodal pattern within 0–0.48 mol/L, with the peak value located in the 0.03–0.12 mol/L interval. Accordingly, five concentration gradients (0–0.24 mol/L) covering the rising, peak and declining stages were selected for the formal in-depth experiment to balance research accuracy and experimental workload. For the mineral treatments, 2 g of Kao and 2 g of Mont were added individually to separate sets of flasks. Cat and Glu were consistently added to all experimental flasks (excluding the CK) at a final concentration of 0.06 mol/L each, and these substrates were co-incubated with Gly at five specific concentrations: 0 mol/L (Gly0), 0.03 mol/L (Gly0.03), 0.06 mol/L (Gly0.06), 0.12 mol/L (Gly0.12), and 0.24 mol/L (Gly0.24). For CK, 2 g of Kao and 2 g of Mont were added separately to phosphate-buffered saline without any organic substrates (Cat, Glu, or Gly). All reactions were conducted in triplicate.

The liquid shake-flask culture was initiated under a sterile and dark-controlled environment at 28°C, which is consistent with the average temperature of temperate agricultural soils, with a rotation speed of 150 rpm. Throughout the culture period, a 2 mL aliquot of the supernatant was extracted at predetermined times (0, 3, 6, 18, 28, 48, 76, 124, 172, 240, and 360 h) based on the pre-experimental kinetics. Each aliquot was centrifuged at 16,000 rpm for 5 minutes, and 1 mL of the supernatant was diluted to 25 mL for UV-Vis analysis. TOC content was measured using a Vario TOC cube analyzer (Elementar, Germany).

### 2.3. Extraction and purification of HLA

At 0, 18, 48, 76, 124, 172, 240, and 360 h, a 10 mL aliquot of the reaction suspension was withdrawn and centrifuged at 16,000 rpm for 10 min to separate the mineral precipitate and the dark-brown supernatant containing dissolved HLS. The obtained supernatant was acidified to pH 1.0 with 1.0 mol/L HCl and equilibrated for 24 h to fully precipitate HLA. Complete HLA precipitation was confirmed in two steps: (1) no additional flocculation was observed when the supernatant was re-acidified to pH 0.8, and (2) UV-Vis spectroscopy showed no absorbance at 280 nm (a characteristic wavelength for HLA) in the post-centrifugation supernatant.

Following confirmation, the mixture was subjected to another high-speed centrifugation (16,000 rpm for 10 min): the clear supernatant (fulvic-like acid, FLA) was neutralized with 0.1 mol/L NaOH and diluted to 25 mL; the precipitate (crude HLA) was dissolved in 0.1 mol/L NaOH at 60°C, neutralized, and diluted to 25 mL. C contents of HLA and FLA (C_HLA_ and C_FLA_) were measured via TOC analysis. After 360 h, the remaining HLA was purified by rinsing with 150 mL of 6% HCl-6% HF to remove inorganic impurities, magnetically stirred for 24 h, centrifuged (16,000 rpm for 10 min), freeze-dried, ground, and sieved through a 0.01 mm mesh for elemental analysis and FTIR analysis. To evaluate the extraction recovery and ensure comparability of HLA fractions across different mineral systems, standard addition recovery experiments were performed: a known amount of purified HLA standard was added to the mineral suspension before extraction, and the recovery rate was calculated based on the measured C content. C mass balance analysis was further conducted by summing supernatant TOC, C_HLA_, C_FLA_, and mineral-associated organic C (MAOC), with the total C recovery calculated as the ratio of measured total C to theoretical total C input.

### 2.4. Mineral sample preparation

At 360 h, the mineral precipitates (post-HLA separation) were rinsed with 30% H_2_O_2_ to eliminate physically adsorbed HLS; the resulting solid underwent identical pretreatment (freeze-drying, thorough grinding, and sieving through a 0.01-mm mesh) to obtain the rinsed mineral sample, which was subjected to the same set of analyses. MAOC of the rinsed mineral samples was determined via dry combustion method using a Vario TOC cube analyzer (Elementar, Germany). Sequential desorption experiments were conducted to quantify the binding stability of mineral-bound organic matter: minerals were sequentially extracted with ultrapure water (3 times), 0.1 mol/L Na_4_P_2_O_7_·10H_2_O solution, and 0.1 mol/L NaOH solution, with TOC content in each desorption fraction measured to distinguish loosely bound, moderately bound, and stably bound organic C pools.

### 2.5. Analysis method

The absorbances of diluted aliquots of the supernatant and diluted liquid HLA samples were determined at 465 nm (E_4_) and 665 nm (E_6_) using a UV-visible spectrophotometer (TU-1900; Beijing Purkinje General Instrument Co., Ltd., Beijing, China). The E_4_/E_6_ ratio was calculated as the ratio of the two absorbance values, which characterizes the degree of aromatic condensation and molecular structure complexity of HLS. The TOC contents of the supernatant, HLA solution, and FLA solution were measured using a Vario TOC cube analyzer (Elementar, Germany) to quantify the accumulation of organic C associated with HLS formation. Subsequently, the C_HLA_/C_FLA_ ratio was calculated. The elemental compositions (C, H, O, and N) of the purified HLA samples were determined using an elemental analyzer (PE 2400II CHNS/O; Perkin-Elmer, Waltham, MA, USA). The HLA and mineral precipitates, obtained via the treatment procedure specified in Section 2.2.3, were analyzed by FTIR spectroscopy on an FTIR-850 instrument (Tianjin Gangdong Technology Development Co., Ltd., Tianjin, China), with spectra recorded across the wavenumber region of 400–4000 cm^-1^. The acquired spectra were processed using FTIR 850 software and visualized with OriginPro 2026 software (OriginLab Corporation, Northampton, MA, USA). For quantitative functional group analysis, second-derivative spectra and Gaussian peak deconvolution were performed on FTIR spectra in the 1800–1000 cm^-1^ region using OriginPro 2026 software. The relative contents of aromatic C = C, aliphatic C–H, and polysaccharide C–O functional groups were calculated as the percentage of each deconvoluted peak area relative to the total peak area of the fitted region, with all fitting coefficients (R^2^) above 0.995.

### 2.6. Statistical analysis

Data organization and preliminary processing were conducted using Microsoft Excel 2021. For spectral plotting and data fitting, OriginPro 2026 software was employed: the temporal dynamics of the E_4_/E_6_ ratio were fitted with the Logistic5 model (Equation 1), while the temporal changes in TOC content were simulated using the Boltzmann model (Equation 2).

**Equation 1: Logistic 5 model for E**_**4**_**/E**_**6**_ **ratio fitting**


$$\begin{array}{c} y=A_{min}+\frac{A_{max}-A_{min}}{{\left(\text{1}+{\left(\frac{x_{\text{0}}}{x}\right)}^{\text{h}}\right)}^{s}} \end{array}$$ (1)

**Equation 2: Boltzmann model for TOC temporal dynamics fitting**


$$\begin{array}{c} \text{Y}={\text{A}}_{\text{2}}+\frac{{\text{A}}_{\text{1}}-{\text{A}}_{\text{2}}}{\text{1}+{\text{e}}^{\left(\text{x}-{\text{x}}_{\text{0}}\right)/\text{dx}}} \end{array}$$ (2)

Statistical analysis was performed using SPSS 27.0 software. First, ANOVA was used to assess the overall differences among all treatment groups. Subsequently, the LSD method was applied for post-hoc pairwise comparisons to clarify the significance of differences between specific groups. A significance level of *p* < 0.05 was adopted for all statistical tests.

For nonlinear kinetic model fitting, residual normality tests and 95% confidence intervals of all fitted parameters were performed to exclude potential overfitting. Alternative kinetic models (first-order kinetic, second-order kinetic, and Elovich models) were also compared, and the optimal model was selected based on the highest adjusted R^2^ and lowest Akaike information criterion (AIC) value.

PLS-SEM was conducted using the plspm package in R 4.5.1 to reveal the pathways linking Gly addition to humification processes and C sequestration in the Maillard reaction-mineral system. The overall model goodness-of-fit (GOF) was calculated to evaluate global model performance. Sensitivity analysis of latent variable indicators was conducted to verify model robustness, and the significance of path coefficients was tested via 1000 bootstrap resamples. All basic data visualization was finished using OriginPro 2026. The latent variables and their corresponding observed indicators of the PLS-SEM model are listed in Table 2.

**Table 2:** Latent variables and corresponding observed indicators of the PLS-SEM model.

Model variables	Observed indicators
Gly addition concentration	Gly concentration
HLS properties	E_4_/E_6_ (supernatant), TOC (supernatant)
Mineral-organic coupling	FTIR (mineral) 1090 cm^-1^
HLS accumulation	C_HLA_, C_HLA_/C_FLA_
C sequestration	C_HLA_, TOC (Supernatant)
HLS molecular structure	E_4_/E_6_ (HLA), C/N, O/C, FTIR (HLS) 1630 cm^-1^
HLS structural complexity	E_4_/E_6_ (HLA)

## 3. Results

### 3.1. E_4_/E_6_ Ratio and TOC in the supernatant

The E_4_/E_6_ ratio serves as an indicator of the degree of aromatic condensation of dissolved HLS, with a higher ratio reflecting a lower degree of aromatic condensation and a comparatively less complex molecular structure [15]. Notably, an increase in supernatant E_4_/E_6_ ratio did not indicate reduced HLA aromatic condensation; instead, it reflected the preferential adsorption of highly aromatic, high-molecular-weight HLS fractions onto mineral surfaces, leaving relatively simple-structured low-molecular-weight components in the aqueous phase. As shown in Fig 1, the E_4_/E_6_ ratios of both Mont- and Kao-mediated systems exhibited distinct temporal dynamics during the reaction process: an overall decreasing trend with slight fluctuations at the initial stage, followed by gradual stabilization in the later period. The Logistic5 model achieved excellent fitting effects for these temporal trends (Table 3). All fitted models passed residual normality tests with randomly distributed residuals and no systematic deviation. The 95% confidence intervals of *A*_*max*_ and *x*_*0*_ parameters were narrow (relative width < 5% for all treatments), indicating high parameter stability. Compared with alternative first-order and Elovich models, the Logistic5 model improved the adjusted R^2^ by 0.031–0.076 and reduced AIC by 18.4–32.7, confirming that the high determination coefficients arose from the inherent S-shaped kinetic characteristics of HLS structural evolution rather than overfitting. *A*_*max*_ directly reflected the dynamic evolution endpoint of the molecular structure of the supernatant. In the Mont-mediated Maillard reaction system, *A*_*max*_ showed a continuous upward trend with increasing Gly dosage. As the Gly concentration increased from 0 to 0.24 mol/L, *A*_*max*_ gradually increased from 5.36 to 13.31008, corresponding to a relative increase of 148.3%. The Kao-mediated Maillard system exhibited the same variation trend in *A*_*max*_: *A*_*max*_ increased from the initial value of 3.95734 to 14.29765, achieving an overall relative increase of 261.3%.

**Table 3:** Logistic5 model fitting parameters for variation of the E_4_/E_6_ ratio in the supernatant mediated by Kao and Mont with varying Gly concentrations.

Mineral types	Treatment	Polynomial Fitting of E_4_/E_6_ $y=A_{min}+\frac{A_{max}-A_{min}}{{\left(1+{\left(\frac{x_{0}}{x}\right)}^{𝐡}\right)}^{s}}$	Coefficient (R^2^)	A_max_	x_0_
**Mont**	**Gly0**	y = 2 + (5.36 - 2)/(1 + (3.75661/x)^-0.29497^)	0.99803	5.36	3.75661
	**Gly0.03**	y = 3.03987 + (8.95833 - 3.03987)/(1 + (3.87604/x)^-0.62787^)	0.99885	8.95833	3.87604
	**Gly0.06**	y = -2.43232 + (10.49595 + 2.43232)/(1 + (5.52934/x)^-0.48677^)^0.37197^	0.99729	10.49595	5.52934
	**Gly0.12**	y = 2.45327 + (11.39359- 2.45327)/(1 + (11.98035/x)^-0.49978^)^0.70516^	0.99956	11.39359	11.98035
	**Gly0.24**	y = 2.05635 + (13.31008 - 2.05635)/(1 + (9.84153/x)^-0.47641^)^0.76492^	0.99573	13.31008	9.84153
	**CK**	y = 1.06488 + (1.80455 - 1.06488)/(1 + (70.28386/x)^18.29827^)^0.05402^	0.99989	1.80455	70.28386
**Kao**	**Gly0**	y = 2.3997 + (3.95734 - 2.3997)/(1 + (2.45286/x)^-15.55231^)^0.0459^	0.99982	3.95734	2.45286
	**Gly0.03**	y = -2.63678 + (8.59768 + 2.63678)/(1 + (2.47203/x)^-0.34654^)^0.35129^	0.99744	8.59768	2.47203
	**Gly0.06**	y = -2.99611 + (12.501 + 2.99611)/(1 + (3.00423/x)^-0.45807^)^0.38048^	0.99569	12.501	3.00423
	**Gly0.12**	y = 4.26908 + (13.5 - 4.26908)/(1 + (15.00871/x)^-0.73676^)	0.99641	13.5	15.00871
	**Gly0.24**	y = -3.93758 + (14.29765 + 3.93758)/(1 + (4.17139/x)^-0.34059^)^0.38868^	0.99708	14.29765	4.17139
	**CK**	y = 0.4229 + (1.28994 - 0.4229)/(1 + (312.14561/x)^-6.15273^)^13.32312^	0.99954	1.28994	312.14561

Note: A_min_, minimum E_4_/E_6_ ratio; *A*_*max*_, maximum E_4_/E_6_ ratio; *x*_*0*_, critical time at the inflection point of the E_4_/E_6_ ratio; *h*, rate coefficient of E_4_/E_6_ ratio variation; *s*, shape parameter of the fitting curve; and R^2^, coefficient of determination.

**Fig 1:**
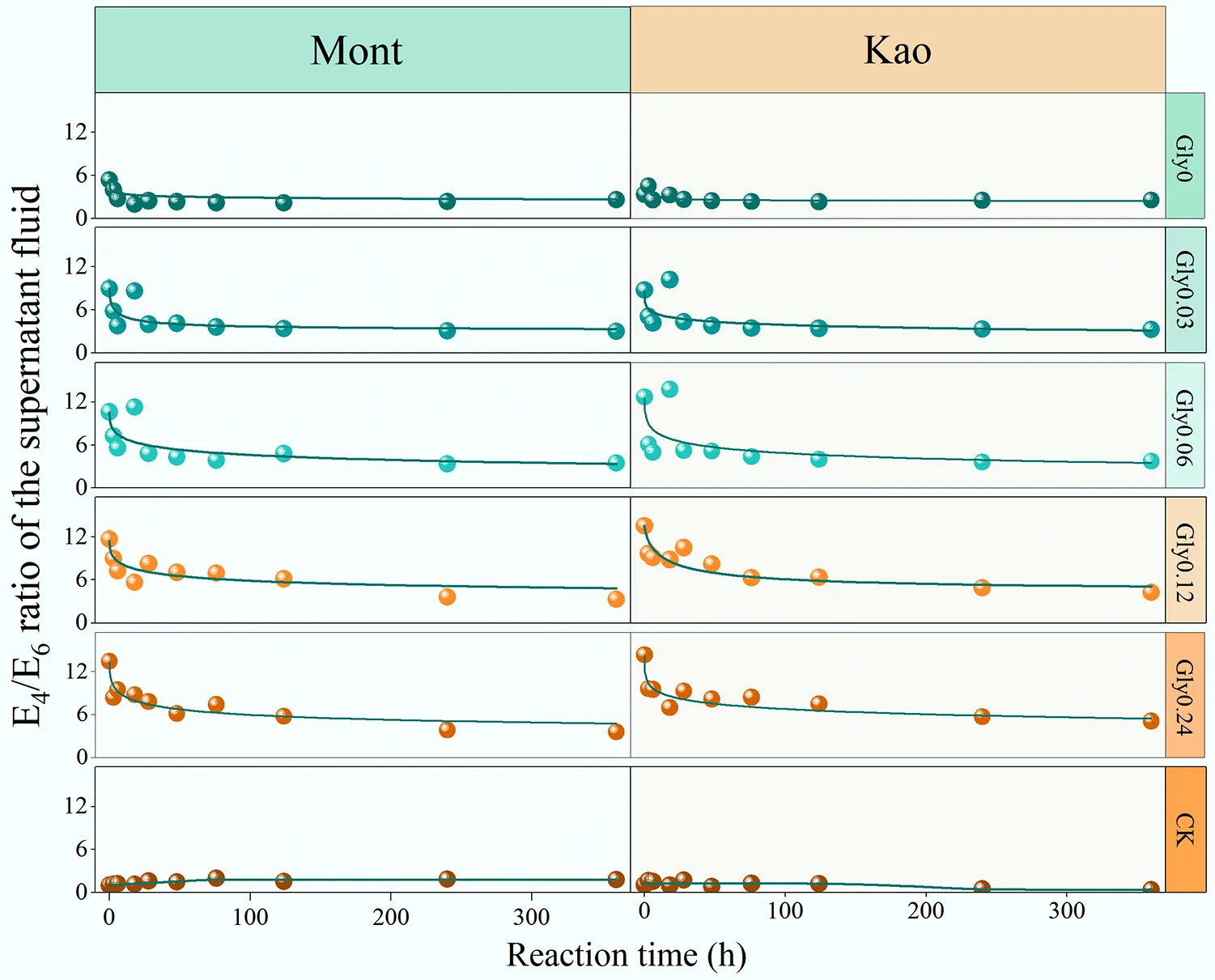
Variation in the E_4_/E_6_ ratio in the supernatant mediated by Kao and Mont under varying Gly concentrations. Note: Data points represent the mean values of the experimental measurements (*n* = 3); error bars indicate the corresponding standard deviations; and solid curves depict the best-fit results generated using the five-parameter logistic (Logistic5) model.

*X*_*0*_, the critical time point at which the E_4_/E_6_ ratio changed most drastically, was positively correlated with the critical threshold of HLS structural transformation. In both Mont- and Kao-mediated Maillard systems, *x*_*0*_ exhibited a “first increasing and then decreasing” trend with increasing Gly concentration, reaching a peak at a concentration of 0.12 mol/L. A quantitative comparison revealed that, except for the 0.12 mol/L concentration group, *x*_*0*_ in the Mont system was larger than that in the Kao system: in the Mont group, *x*_*0*_ increased from 3.75661 at 0 mol/L to 11.98035 at 0.12 mol/L and then decreased to 9.84153 at 0.24 mol/L. In the Kao group, *x*_*0*_ increased from 2.45286 at 0 mol/L to 15.00871 at 0.12 mol/L; however, when the concentration further increased to 0.24 mol/L, *x*_*0*_ dropped sharply to 4.17139, demonstrating more obvious fluctuation characteristics.

As illustrated in Fig 2, the temporal dynamics of TOC content in the supernatant across all treatment groups were fitted with the Boltzmann model, and high R^2^ values (Table 4, R^2^ > 0.999) verified the validity of the fitting. Residual analysis showed no systematic bias in fitting results, and the 95% confidence intervals of *A*_*1*_, *A*_*2*_, and *x*_*0*_ parameters all had relative deviations below 4.5%. Model comparison confirmed that the Boltzmann model outperformed other tested kinetic models in describing TOC accumulation dynamics, with adjusted R^2^ 0.024–0.069 higher than the first-order kinetic model. These results demonstrated that the near-unity R^2^ values reflected the typical sigmoidal pattern of Maillard reaction C accumulation, rather than statistical overfitting. As shown in Table 4, both *A*_*1*_ and *A*_*2*_ showed an upward trend with increasing Gly concentration; however, distinct disparities in the kinetic stability of TOC profiles were observed between the two mineral groups. As fitting parameters of the Boltzmann equation employed for kinetic modeling of Maillard reaction, *A*_*1*_ and *A*_*2*_ denoted the maximum and minimum asymptotic values of TOC in the reaction system, respectively; these parameters were directly correlated with the final browning degree of Maillard reaction and the accumulation extent of intermediate products. For the Mont group, *A*_*2*_ increased from 7.95639 to 16.10488, corresponding to a 102.41% increase, whereas *A*_*1*_ increased from 11.66669 to 21.79003 with an 86.77% increase. In the Kao group, *A*_*2*_ climbed from 8.51308 to 14.80251, representing a 73.9% increase, while *A*_*1*_ increased from 13.02144 to 21.43943, with a 64.7% rise. Notably, the growth rate of *A*_*2*_ in the Mont group exceeded that in the Kao group, and the same trend was observed for *A*_*1*_, with the Mont group exhibiting a higher growth rate than the Kao group.

**Table 4:** Boltzmann model fitting parameters for temporal variations in TOC in the supernatant under Kao and Mont mediation with different Gly concentrations.

Mineral types	Treatment	Polynomial Fitting of TOC $𝐘={𝐀}_{2}+\frac{{𝐀}_{1}-{𝐀}_{2}}{1+{𝐞}^{\left(𝐱-{𝐱}_{0}\right)/𝐝𝐱}}$	Coefficient (R^2^)	A_1_	A_2_
**Mont**	**Gly0**	y = 7.95639 + (11.66669 - 7.95639)/(1 + exp((x - 5.94524)/0.11938))	0.99985	11.66669	7.95639
	**Gly0.03**	y = 8.02995 + (11.69578 - 8.02995)/(1 + exp((x - 94.52046)/6898.58975))	0.99976	11.69578	8.02995
	**Gly0.06**	y = 9.38997 + (14.94919 - 9.38997)/(1 + exp((x - 4.49915)/1.4762))	0.99978	14.94919	9.38997
	**Gly0.12**	y = 11.13537 + (17.30075 - 11.13537)/(1 + exp((x + 14.30686)/18))	0.99967	17.30075	11.13537
	**Gly0.24**	y = 16.10488 + (21.79003 - 16.10488)/(1 + exp((x - 3.05843)/0.04688))	0.99986	21.79003	16.10488
	**CK**	y = 0.79539 + (0.35081 - 0.79539)/(1 + exp((x - 28.64874)/4.16844))	0.9996	0.35081	0.79539
**Kao**	**Gly0**	y = 8.51308 + (13.02144 - 8.51308)/(1 + exp((x - 4.77107)/0.80871))	0.99979	13.02144	8.51308
	**Gly0.03**	y = 10.08899 + (14.94524 - 10.08899)/(1 + exp((x + 23.01088)/18))	0.9992	14.94524	10.08899
	**Gly0.06**	y = 10.04109 + (14.81755 - 10.04109)/(1 + exp((x - 3.99742)/1.23821))	0.99947	14.81755	10.04109
	**Gly0.12**	y = 11.94925 + (17.41913 - 11.94925)/(1 + exp((x - 5.18784)/1.20051))	0.99979	17.41913	11.94925
	**Gly0.24**	y = 14.80251 + (21.43943 - 14.80251)/(1 + exp((x - 4.78769)/0.71463))	0.99967	21.43943	14.80251
	**CK**	y = 0.67027 + (0.85514 - 0.67027)/(1 + exp((x - 243.05034)/3.64232))	0.99973	0.85514	0.67027

Note: *A*_*1*_ and *A*_*2*_ represent the maximum and minimum TOC contents, respectively; *x*_*0*_, inflection point time (peak reaction rate); *d*_*x*_, rate of TOC change; and R^2^, coefficient of determination.

**Fig 2:**
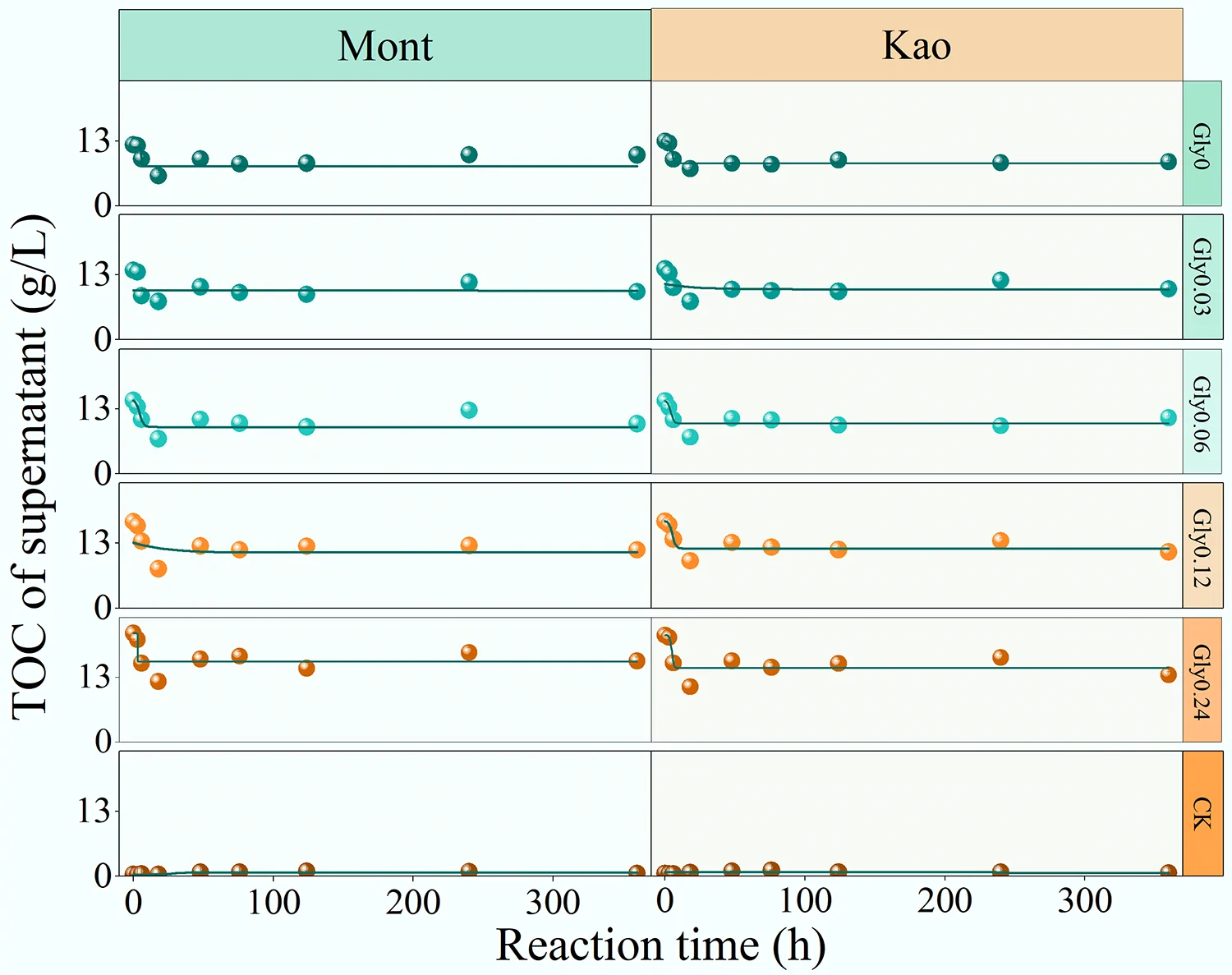
Variations in TOC content of the supernatant mediated by Kao and Mont under varying Gly concentrations.

### 3.2. C of humic-like acid (C_HLA_), C of humic-like acid to C of fulvic-like acid (C_HLA_/C_FLA_) ratio, and E_4_/E_6_ ratio of HLA extracted from the dark-brown residue

As shown in Fig 3, distinct temporal variations in the C_HLA_ were observed during Maillard reaction mediated by two clay minerals. In the early reaction stage (0–76 h), C_HLA_ concentrations in both mineral systems fluctuated stably without continuous accumulation. For the Mont group, C_HLA_ content fluctuated in the range of 0.81–0.90 g/kg (calculated as the average value of all Gly treatments at each time point), while the Kao group maintained a relatively higher C_HLA_ level with a variation range of 1.12–1.44 g/kg.

**Fig 3:**
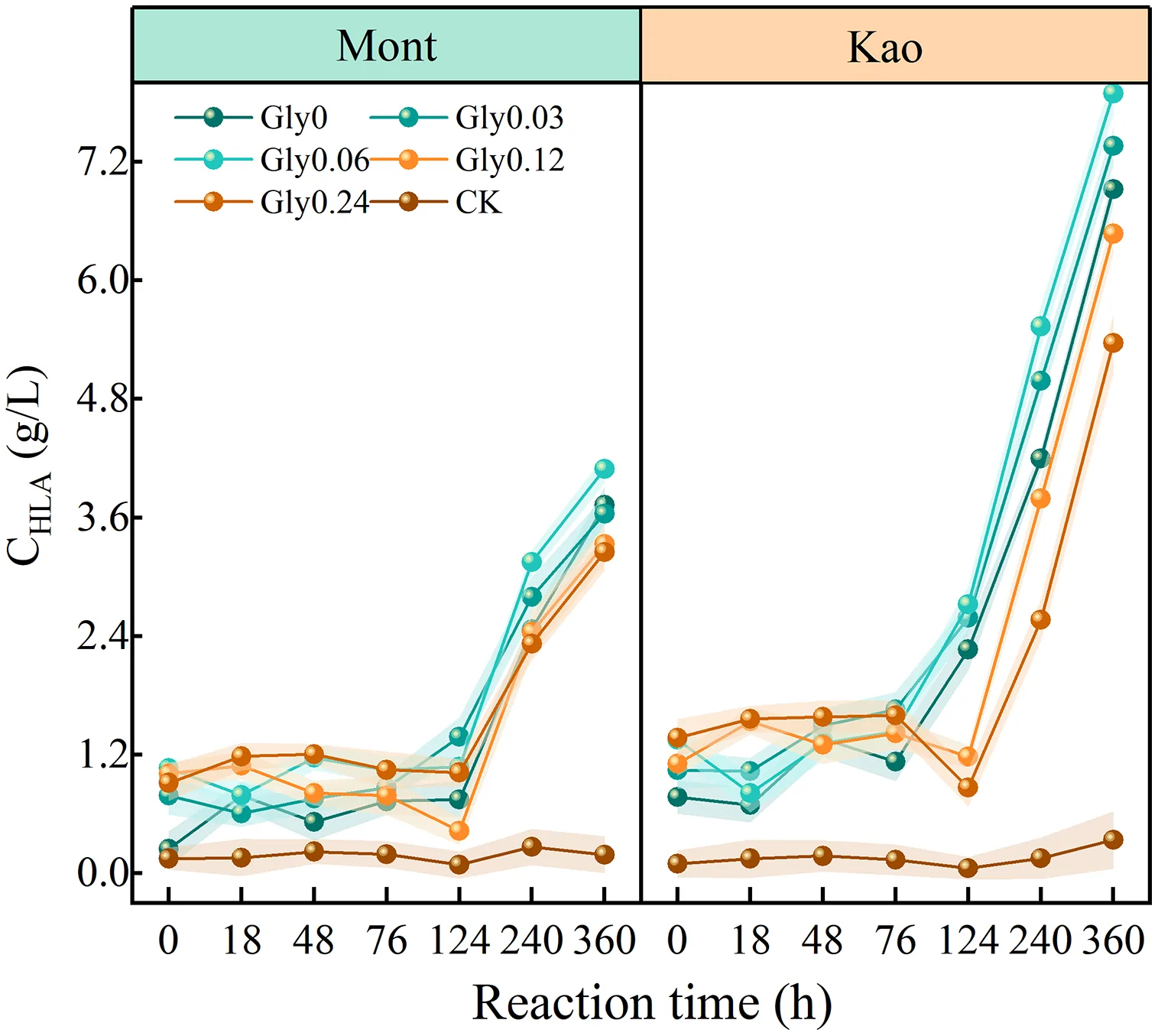
C_HLA_ extracted from the dark-brown residue during Maillard reaction is plotted as a function of Kao, Mont, and varying concentrations of Gly.

The late stage (124–360 h) was identified as the critical period for rapid C_HLA_ accumulation. In the Mont system, C_HLA_ sharply increased from 0.93 g/kg at 124 h to 3.60 g/kg at 360 h. The Kao group exhibited a much more pronounced increment, with C_HLA_ rising from 1.92 g/kg to 6.80 g/kg over the same period; its growth rate in the late reaction stage was markedly higher than that in the early stage. Throughout the whole reaction period, the Kao-mediated system retained a higher C_HLA_ content at all time points, demonstrating persistent superiority in promoting HLA formation. In terms of Gly dosage effects, the 0.06 mol/L Gly treatment exerted the optimal promotion effect on C_HLA_ accumulation in both mineral systems, whereas high Gly concentrations presented a weakened promotion capacity. The optimal Gly concentration of 0.06 mol/L was cross-validated by multiple independent indicators: C_HLA_ content, C_HLA_/C_FLA_ maturity ratio, mineral-organic coupling FTIR signal, and HLA aromatic condensation degree all reached their optimal values at this concentration, showing high consistency across C accumulation, molecular maturity and mineral interaction levels. Kinetic analysis further confirmed that the 0.06 mol/L treatment achieved the highest maximum reaction rate while maintaining high final C_HLA_ accumulation, representing the best balance between reaction efficiency and product yield. The average C_HLA_ content of the Gly0.06 treatment reached 1.77 g/kg in the Mont group, which was significantly higher than that of other dosage groups. Similarly, the Kao-Gly0.06 group obtained the maximum C_HLA_ value of 3.00 g/kg among all experimental treatments. Extraction recovery tests showed that the average HLA recovery rates were 92.7 ± 2.1% for the Mont system and 91.8 ± 2.5% for the Kao system, with no significant difference between the two mineral groups (*p* > 0.05). C mass balance analysis further verified that the total C recovery of all treatments ranged from 94.2% to 97.8%, indicating negligible C loss during the extraction process. These results confirmed that the extraction efficiency of HLA was independent of mineral type, and the C_HLA_ data across Mont and Kao systems were directly comparable.

C_HLA_/C_FLA_ ratio, a core indicator reflecting the maturity of HLS [16], exhibited divergent evolutionary trends between the two mineral systems (Fig 4). From 0 to 360 h, the mean C_HLA_/C_FLA_ ratio of all treatments in the Kao system increased continuously from 0.09 to 0.54, with a total relative increase of 492.8%, with no stationary phase observed during the reaction process. In contrast, the Mont system showed a slow growth rate in the C_HLA_/C_FLA_ ratio: the average value increased slightly from 0.08 to 0.20, representing a total relative increase of only 160.5%. Notably, the C_HLA_/C_FLA_ ratio of the Mont system reached a plateau after 240 h, with negligible subsequent variation.

**Fig 4:**
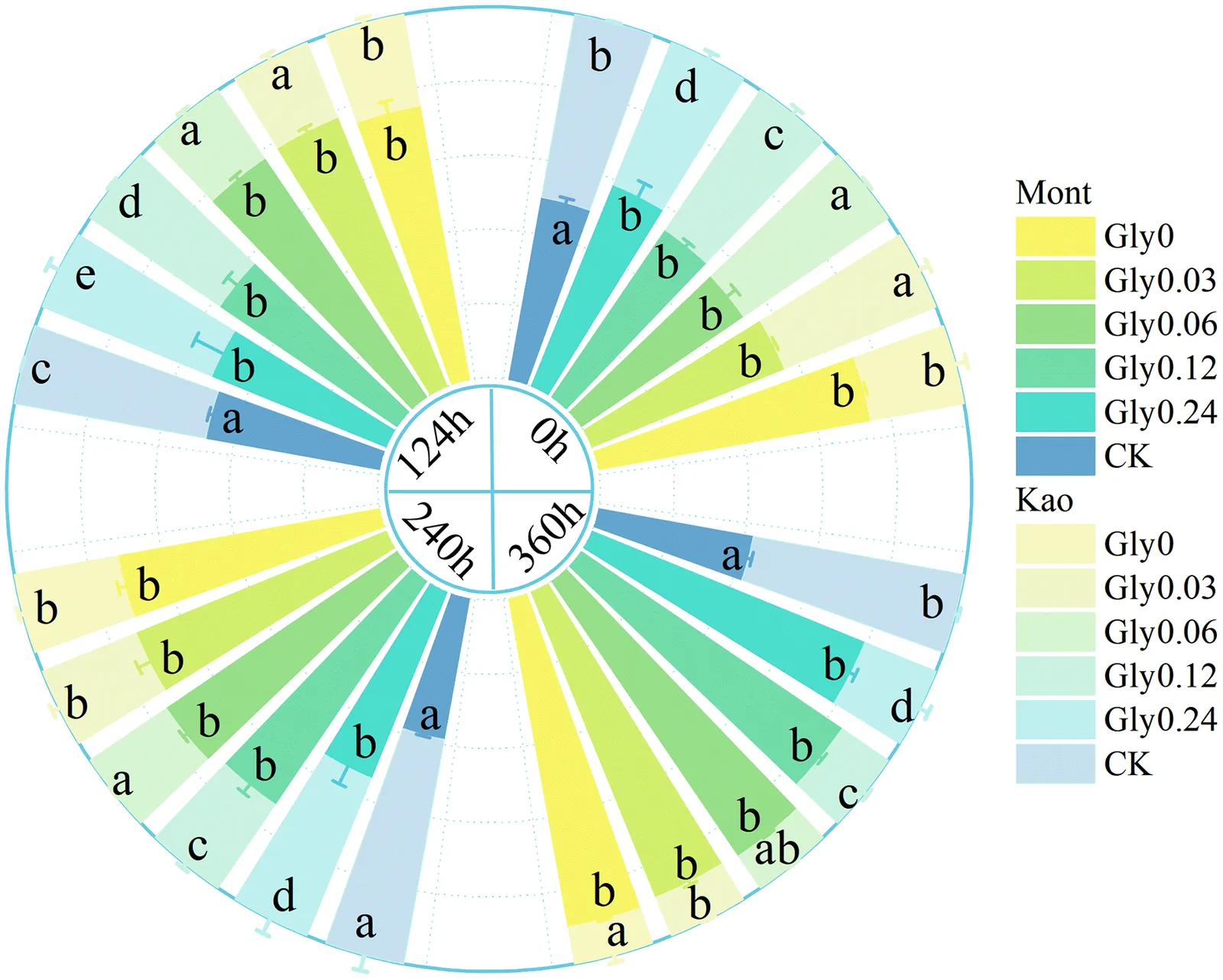
C_HLA_/C_FLA_ ratio of the dark-brown residue as influenced by mineral types and Gly concentrations. Note: Different lowercase letters denote significant differences across treatments under the same incubation time (*p* < 0.05; *n* = 3). Error bars represent standard deviations.

Mont exhibited inferior performance in mediating the Maillard reaction to form complex HLA compared to Kao. Compared with the Gly0, Gly0.03, Gly0.06, Gly0.12, and Gly0.24 treatment groups of the Kao group, the average E_4_/E_6_ value of the Mont group over the entire period was increased by 3.6%, 35.8%, 19.1%, 16.8%, and 22.7%, respectively, indicating that Mont-mediated HLA had an overall lower aromatic condensation degree and simpler structure. As illustrated in Fig 5, the E_4_/E_6_ ratio increased rapidly in the early stage of the reaction, peaked at 76 h, and then gradually decreased.

**Fig 5:**
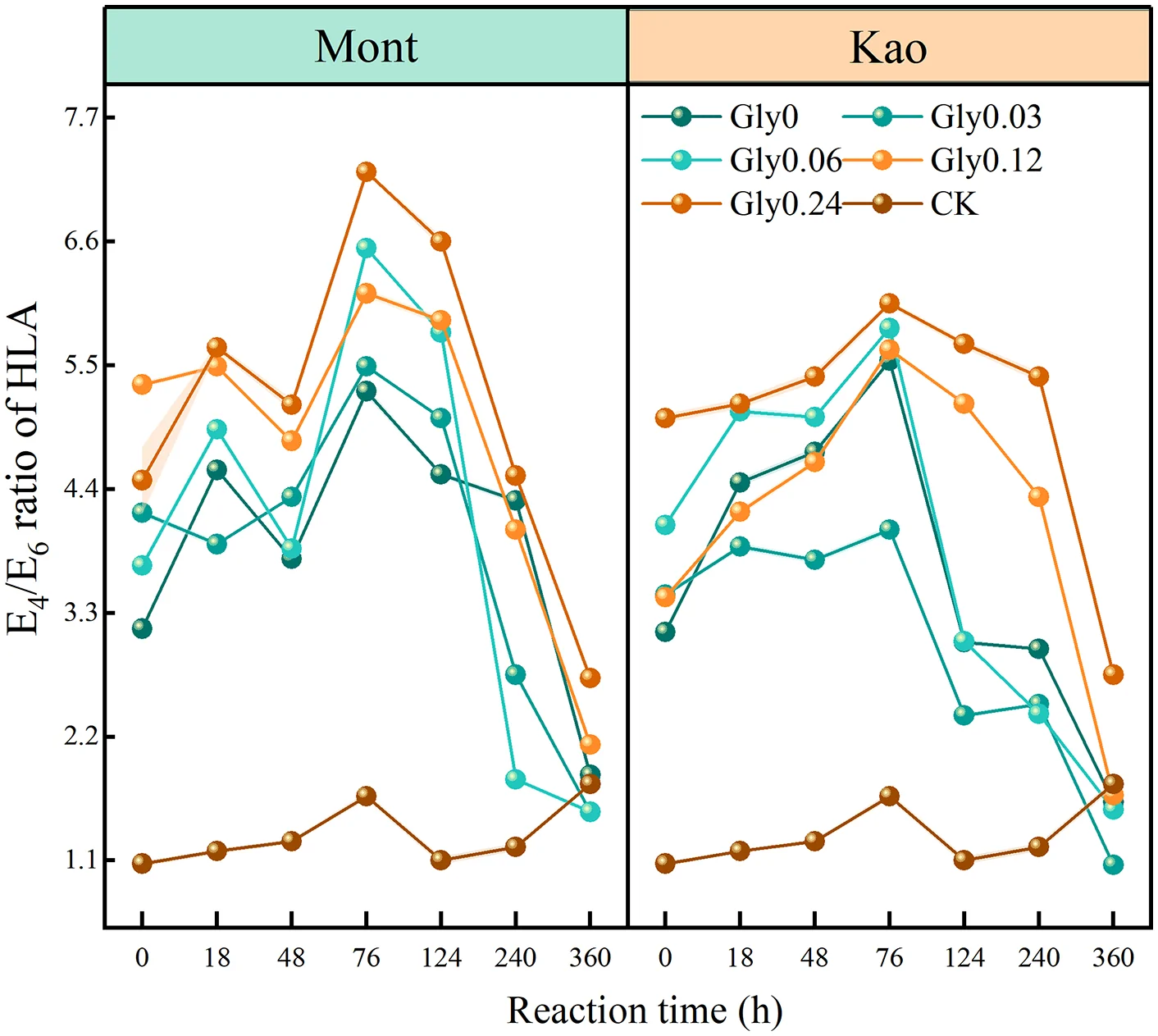
Variations in the E_4_/E_6_ ratio of HLA extracted from the dark-brown residue under mediation of Kao and Mont with different Gly concentrations.

### 3.3. Atomic molar ratios and FTIR spectra of HLA extracted from the dark-brown residue

The H/C ratio was negatively correlated with aromaticity, with lower values indicating a higher degree of aromatic condensation [17]. The C/N ratio reflected the relative abundance of C and N in HLA molecules; a reduction in the C/N ratio suggested the preferential enrichment of N within stable, N-rich structural domains [18]. The O/C ratio served as an indicator of oxidation degree and molecular polarity, with higher values corresponding to greater oxidation and enhanced polarity [18]. The data presented in Table 5 demonstrated that, for the H/C ratio, values in all Mont-treated groups at different Gly concentrations were consistently higher than those in the corresponding Kao-treated groups. Regarding the C/N ratio, values in the Mont system exhibited a gradual decreasing trend from 43.5 to 8.95 with increasing Gly concentration. In the Kao system, although a slight fluctuation was observed at a Gly concentration of 0.06 mol/L (where the C/N ratio increased from 12.7 to 15.8), the C/N ratio still showed an overall decreasing tendency from 42.4 to 8.37 as Gly levels increased. With respect to the O/C ratio, both Mont and Kao groups displayed a pattern of initial increase followed by a decrease, with the peak value occurring at a Gly concentration of 0.03 mol/L. The maximum O/C ratios were 1.02 for Mont and 1.34 for Kao at this Gly addition level, respectively.

**Table 5:** Atomic molar ratios of HLA extracted from the dark-brown residue after Maillard reaction mediated by Kao and Mont with varying Gly concentrations.

Treatments	Mont			Kao		
	H/C	C/N	O/C	H/C	C/N	O/C
**Gly0**	1.28 ± 0.02c	43.53 ± 0.06a	0.95 ± 0.02b	0.94 ± 0.02d	42.35 ± 0.16a	0.82 ± 0.03c
**Gly0.03**	1.92 ± 0.02a	20.22 ± 0.06b	1.02 ± 0.03a	1.83 ± 0.03a	12.73 ± 0.02b	1.34 ± 0.01a
**Gly0.06**	1.94 ± 0.03a	14.15 ± 0.03c	0.91 ± 0.03c	1.01 ± 0.05c	15.77 ± 0.03c	0.94 ± 0.01b
**Gly0.12**	1.75 ± 0.02b	10.83 ± 0.04d	0.85 ± 0.03d	1.10 ± 0.02b	10.86 ± 0.03d	0.73 ± 0.01d
**Gly0.24**	1.37 ± 0.03c	8.95 ± 0.03e	0.80 ± 0.01e	1.13 ± 0.01b	8.37 ± 0.01e	0.73 ± 0.03d

Fig 6 illustrates the FTIR spectra of HLA extracted from the dark-brown residue obtained under different Gly concentrations. The absorption band at approximately 3419–3443 cm^-1^ was assigned to the stretching vibration of –OH groups derived from phenolic or carboxyl moieties in HLA [19]. The peak near 1621–1639 cm^-1^ corresponded to the stretching vibration of aromatic C = C bonds in HLA [13], which is formed through the aromatization of HLA during the Maillard reaction. The absorption peaks at around 1384–1402 cm^-1^ were attributed to the symmetric bending vibration of C–H bonds in methyl groups [20]. Meanwhile, the signals in the range of 1081–1103 cm^-1^ were associated with the C–O stretching vibration of polysaccharides [9].

**Fig 6:**
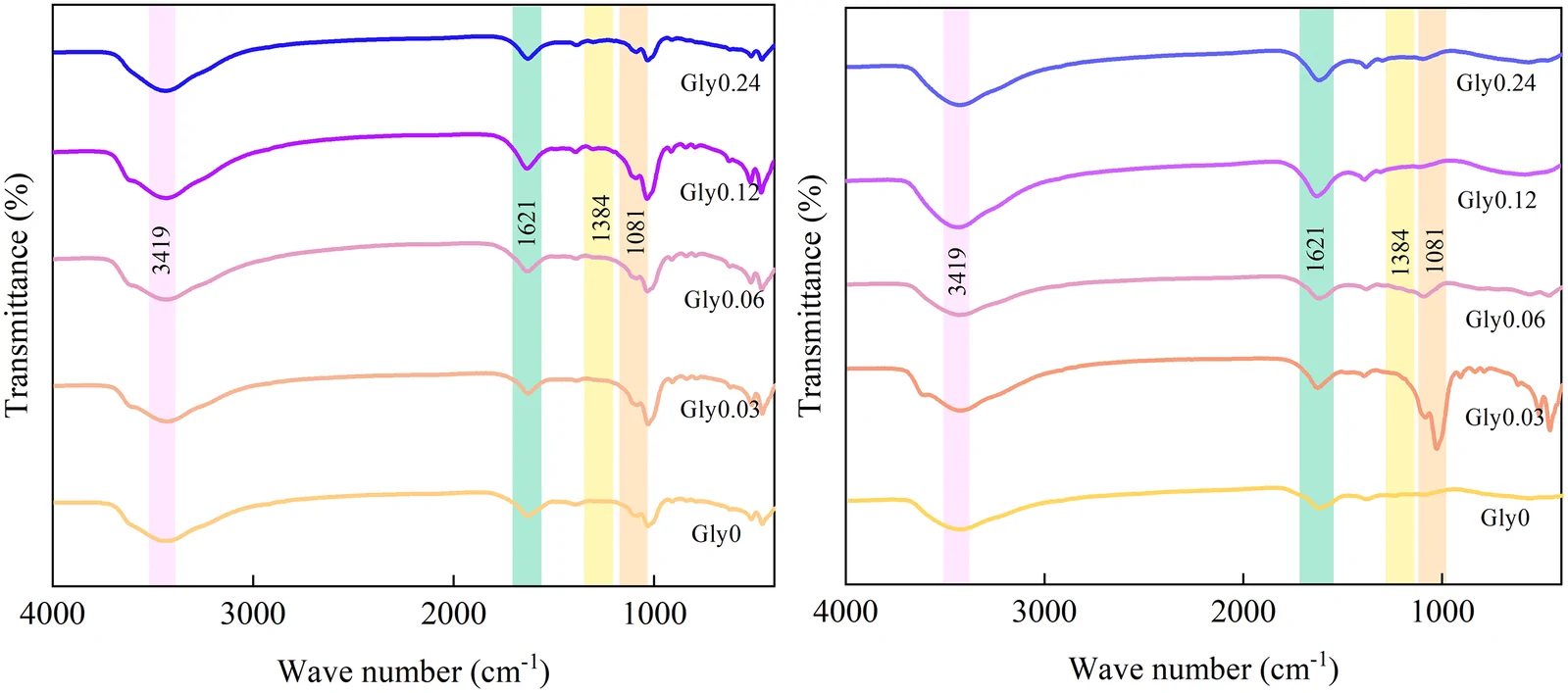
FTIR spectra of HLA extracted from the dark-brown residue generated by Maillard reaction.

With respect to FTIR spectral characteristics, the following regular patterns were observed in Table 6: At the wavenumber ranges of 3419–3443 cm^-1^ and 1621–1639 cm^-1^, the relative peak intensities of the Mont-treated groups at all Gly concentrations were consistently higher than those of the Kao-treated groups in a one-to-one comparison. Conversely, at 1384–1402 cm^-1^, the relative peak intensities of the Kao-treated groups across all Gly concentrations were greater than those of the Mont-treated groups under the corresponding conditions. In the region of 1081–1103 cm^-1^, the fluctuation range of the Kao-treated groups (0.5 to 5.99) was considerably wider than that of the Mont-treated groups (0.91 to 1.01). Quantitative peak deconvolution of the 1800–1000 cm^-1^ region further revealed the dynamic changes in HLA functional groups with Gly concentration. For both mineral systems, the relative proportion of aromatic C = C groups (1621–1639 cm^-1^) showed a unimodal trend with increasing Gly dosage, peaking at 0.06 mol/L in the Kao system (15.2% of total fitted area) and at 0.12 mol/L in the Mont system (12.8%). The relative content of aliphatic C–H groups (1384–1402 cm^-1^) was consistently higher in Kao-derived HLA, while the proportion of polysaccharide C–O groups (1081–1103 cm^-1^) decreased significantly at high Gly concentrations.

**Table 6:** FTIR relative intensities of ores after Maillard reaction under different treatments as a percentage of the total area.

Treatment		3419–3443(cm^-1^)	1621–1639(cm^-1^)	1384–1402(cm^-1^)	1081–1103(cm^-1^)
		–OH groups from phenolic / carboxyl -OH moieties	aromatic C=C bonds	C–H bonds in methyl groups	C–O stretching vibration of polysaccharides
**Kao**	**Gly0**	86.26a	11.69d	1.30c	0.75
	**Gly0.03**	77.46d	14.76a	1.94a	5.84
	**Gly0.06**	80.57c	12.28c	1.16d	5.99
	**Gly0.12**	84.66b	13.30b	1.54b	0.50
	**Gly0.24**	86.04a	11.73d	1.32c	0.91
**Mont**	**Gly0**	86.77e	11.42a	0.89a	0.92
	**Gly0.03**	89.17b	9.62c	0.30e	0.91
	**Gly0.06**	87.19d	11.47a	0.34d	1.01
	**Gly0.12**	90.55a	8.08d	0.37c	1.00
	**Gly0.24**	88.01c	10.55b	0.50b	0.94

Note: The total area is defined as the sum of the integral areas of four characteristic bands.

### 3.4. FTIR spectra of clay minerals before and after the Maillard reaction

As shown in Fig 7, the FTIR spectra of the two clay minerals exhibited distinct characteristic absorption bands within the tested wavenumber range. A broad absorption peak in the wavenumber range of 3419–3443 cm^-1^ is a typical signature of the stretching vibration of intrinsic structural –OH groups in the clay mineral lattice [21]. The absorption peak at 1621–1639 cm^-1^ is assigned to the bending vibration of H–O–H bonds in physically adsorbed water molecules on the surface and within the interlayer domains of clay minerals [22]. This peak is ubiquitous in the infrared spectra of clay minerals, stemming from their inherent ability to adsorb water molecules in the surface and interlayer regions. In addition, a strong absorption peak at 1081–1103 cm^-1^ corresponds to the asymmetric stretching vibration of Si–O bonds in silicate tetrahedron (SiO_4_) structural units [23].

**Fig 7:**
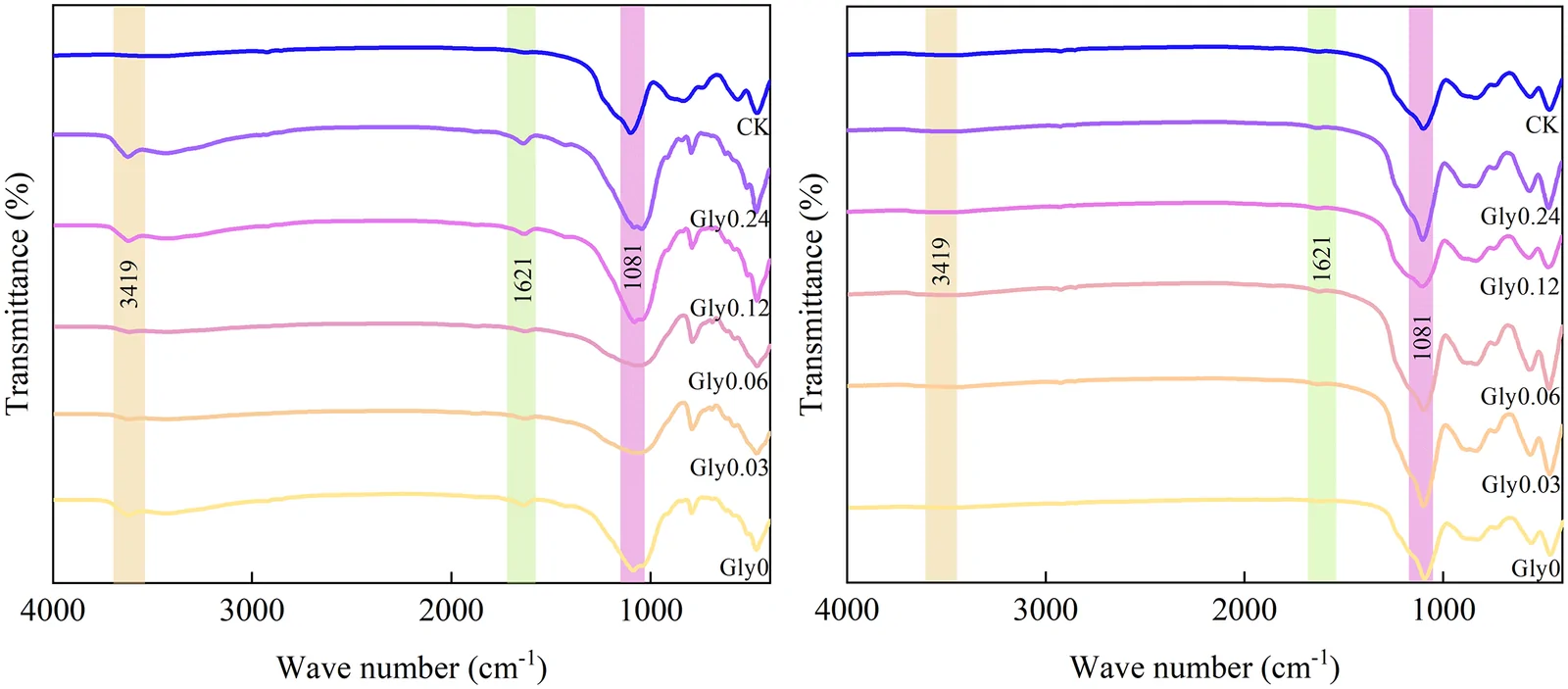
Effects of Kao and Mont at various Gly concentrations on FTIR spectra of the dark-brown residue.

As shown in Table 7, for the Kao group, the characteristic peak of the structural –OH groups presented an overall trend of first increasing and then decreasing, reaching a maximum value of (6.94) at the Gly0.12 treatment. Similarly, the H–O–H absorption peak initially increased and then decreased, with a peak intensity of (0.43) observed at Gly0.06. In contrast, the Si–O framework vibration peak of Kao first decreased and then increased, and reached a minimum value of (61.02) under the Gly0.06 condition. Regarding the Mont group, the H–O–H peak showed an inverse variation trend, which first decreased and then increased and reached a minimum value of (1.06) at Gly0.06. The Si–O framework peak of Mont initially increased and then decreased, yielding a maximum intensity of (77.72) in the Gly0.06 group. In addition, a comparative analysis revealed that the peak intensities of the –OH groups and H–O–H in the Mont group were significantly higher than those in the Kao group throughout the treatments.

**Table 7:** FTIR relative intensity of Kao and Mont after Maillard reaction under different treatments as a percentage of the total area.

Treatment		3419−3443 (cm^-1^) O–H	1621−1639 (cm^-1^) H–O–H	1081−1103 (cm^-1^) Si–O
**Kao**	**Gly0**	2.55e	0.17d	64.93a
	**Gly0.03**	3.32d	0.21c	64.00b
	**Gly0.06**	5.85b	0.43a	61.02d
	**Gly0.12**	6.94a	0.42a	63.43c
	**Gly0.24**	5.24c	0.37b	63.92b
	**CK**	5.05 cd	0.42a	63.98b
**Mont**	**Gly0**	22.29b	1.83a	65.04d
	**Gly0.03**	23.40a	1.63b	67.01c
	**Gly0.06**	19.79c	1.06d	77.72a
	**Gly0.12**	17.83e	1.27c	69.81b
	**Gly0.24**	23.0ab	1.86a	65.69d
	**CK**	18.6d	1.63b	68.83bc

Note: All data are presented as mean values (*n* = 3). Means followed by different lowercase letters within the same mineral group denote statistically significant differences at *p* < 0.05 (one-way ANOVA followed by Tukey’s HSD test).

### 3.5. PLS-SEM

As illustrated in Fig 8, Gly addition positively drove early humification, and mineral–organic coupling acted as a critical intermediate in the subsequent cascade. Mineral interfacial interaction regulated HLS accumulation, and further promoted the sequential evolution of the HLS molecular structure. Both mineral–organic coupling and HLS molecular structure exerted positive effects on potential C stabilization. Meanwhile, HLS molecular structure dominated structural complexity, while the direct effect of C sequestration was weak and non-significant. The overall GOF value of the PLS-SEM model reached 0.82, which was well above the 0.36 threshold for a large effect size, indicating excellent global model fit and strong predictive power. Sensitivity analysis showed that removing any single observed indicator did not change the direction and significance of core structural paths, confirming high robustness of the proposed pathway framework. Overall, PLS-SEM confirmed a stepwise abiotic humification chain, in which exogenous Gly regulation, mineral interface reaction, and HLS molecular maturation jointly determined system C sequestration performance.

**Fig 8:**
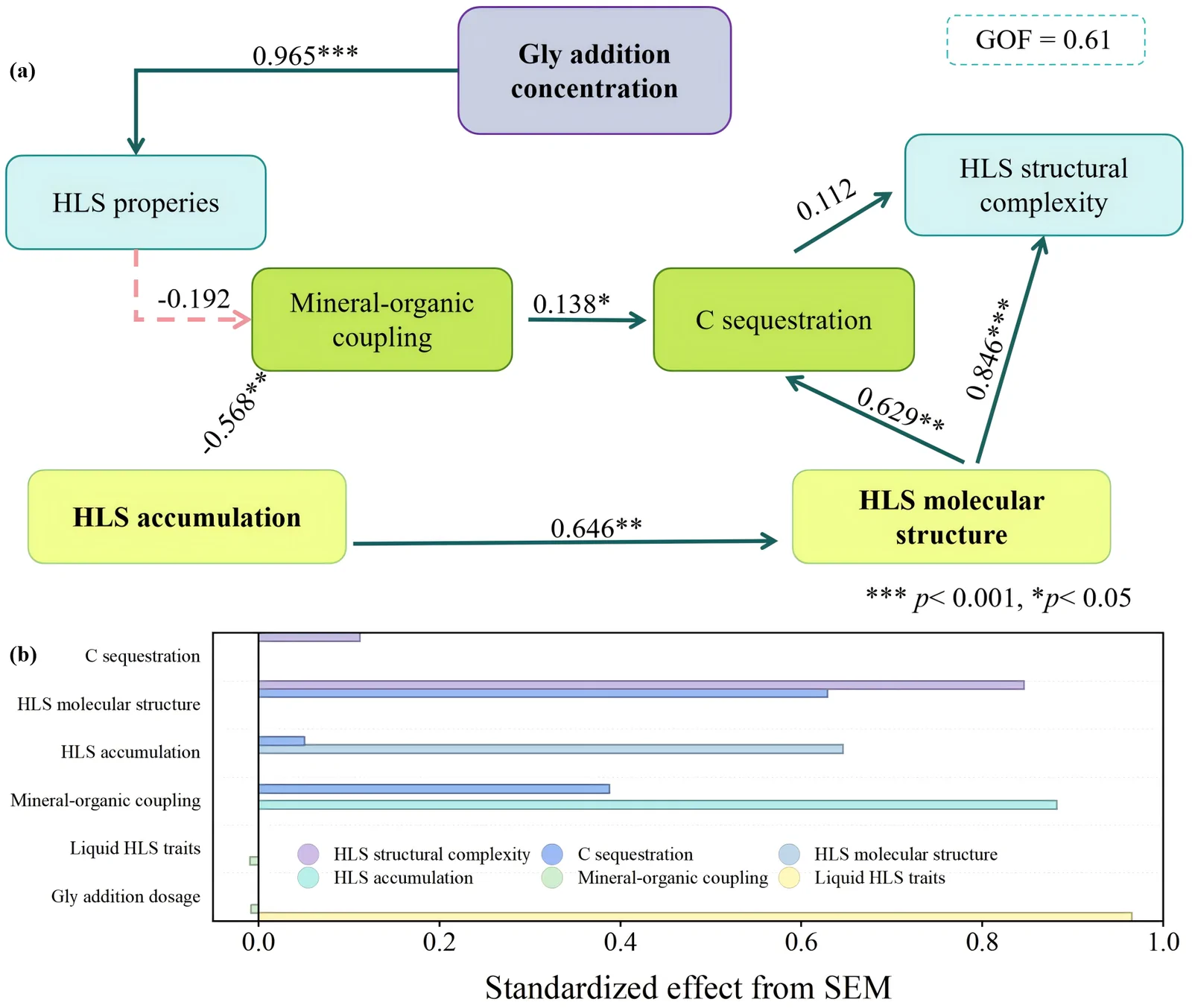
(a) Regulatory mechanism of Gly on humification and C sequestration revealed by PLS-SEM via mineral-organic interactions. (b) Standardized effect sizes of variables in the PLS-SEM for Gly-regulated humification and C sequestration. Note: Latent variables are represented by rounded rectangle shapes. Only structural paths between latent variables are shown; measurement models (latent variables and their corresponding observed indicators) are detailed in the Materials and Methods section. GOF = Goodness of Fit; *** *p* < 0.001, ** *p* < 0.05.

## 4. Discussion

### 4.1. Effect of Gly concentration on E_4_/E_6_ ratio and TOC in the supernatant

In both clay systems, the *A*_*max*_ of the E_4_/E_6_ ratio increased continuously with increasing Gly concentration. This trend was primarily ascribed to the sufficient supply of amino donors, which enhanced the collision frequency of amino-carbonyl groups and further intensified the Maillard reaction [24]. The intensified Maillard reaction facilitated the translocation of highly aromatic condensed HLS from the aqueous phase onto mineral surfaces [25], and simultaneously drove the degradation of reaction intermediates into small oxygenated molecules [26,27]. These two synergistic processes increased the relative proportion of simply structured HLS in the supernatant, thereby resulting in continuous growth of *A*_*max*_.

Compared with Mont, Kao presented a lower initial *A*_*max*_ value but a much higher relative growth rate. Mont exhibited a strong inherent adsorption affinity for high-polymeric HLS [28]. Even under low Gly concentrations, it efficiently adsorbed and removed high-polymeric macromolecular HLS from the supernatant, leaving relatively stable residual components and thereby yielding a higher initial *A*_*max*_ with little room for further elevation along with Gly addition [29]. In contrast, Kao displayed weaker retention capability toward high-polymeric HLS at low Gly dosages, leading to a lower baseline *A*_*max*_ in the liquid phase [30,31]. As the Gly concentration increased, the mineral-mediated Maillard reaction was substantially intensified. This process simultaneously accelerated the degradation of reactive intermediates and the structural transformation of HLS in the supernatant, which ultimately drove a more pronounced rising trend in *A*_*max*_ for the Kao group [25].

Except for the Gly 0.12 mol/L group, the *x*_*0*_ values in the Mont system were larger and more stable than those in the Kao system. Mont immobilized Maillard reaction intermediates via interlayer adsorption, and the steric confinement slowed the structural transformation of HLS in the supernatant—this was reflected by the larger and more stable *x*_*0*_ values in the Mont system (Table 2), because *x*_*0*_ represents the critical time point of drastic HLS structural change, and slower transformation delays this critical point and reduces fluctuations [14]. In contrast, Kao lacked interlayer confinement, and most intermediates remained free or weakly bound to the mineral surface [32], enabling rapid HLS structural evolution and smaller *x*_*0*_. The heterogeneous surface sites and weak binding capacity of Kao led to pulsatile adsorption-desorption of organics, resulting in drastic fluctuations of *x*_*0*_ across Gly gradients [30]. Notably, Gly0.12 represented an optimal dosage, in which amino donor supply matched Mont’s adsorption and catalytic capacity [33]. The interlayer intermediates fully participated in Maillard reaction under interfacial catalysis, accelerating HLS structural transformation and making *x*_*0*_ of Mont slightly lower than that of Kao at this concentration.

Both *A*_*1*_ and *A*_*2*_ of TOC fitting increased with increasing Gly concentration because a higher Gly dosage promoted the generation and accumulation of small-molecule intermediates and provided sufficient substrates for the formation of stable HLS via dehydration, cyclization, and cross-linking condensation [34]. The different growth rates of *A*_*1*_ and *A*_*2*_ between the two systems originated from their differential interfacial regulation. Mont created a local high-concentration microenvironment via interfacial enrichment, and its interlayer cations and edge sites reduced the activation energy of intermediate condensation, favoring the accumulation of liquid-phase intermediates and stable HLS [35,36]. In comparison, Kao only provided mild surface adsorption and weak catalysis without an obvious interfacial concentration effect [37], and the Maillard reaction mainly occurred in the bulk solution with a low reaction efficiency, limiting the increase in TOC-related parameters.

### 4.2. Effect of Gly concentration on C_HLA_, C_HLA_/C_FLA_ and E_4_/E_6_ ratio of HLA

The fluctuating pattern of C_HLA_ in the early stage (0–76 h) was a typical characteristic of the initial stage of the Maillard reaction. The unstable reaction system only produced trace low-molecular-weight intermediates, and no substantial accumulation of HLA precursors was detected [34]. Compared with Mont, Kao possessed stronger surface catalytic activity to activate functional groups of reaction substrates, thereby generating more primary intermediates and nascent humic fractions [38,39]. This difference explained the higher initial C_HLA_ background and wider fluctuation range in the Kao system.

The 124-h period was a critical turning point in the reaction evolution. At the late reaction stage (124–360 h), the synergistic effect between Maillard reaction and mineral interfacial action gradually stabilized. Massive intermediate metabolites underwent continuous dehydration, cyclization, and cross-linking condensation reactions, which accelerated the formation of high-molecular-weight HLA and triggered a sharp increase in C_HLA_ content [27]. The distinct C_HLA_ accumulation patterns between the two minerals were primarily attributed to their unique interfacial properties. Mont could immobilize partial substrates and intermediates within interlayer spaces, and the spatial confinement effect restricted the cross-linking and polymerization of macromolecular intermediates, ultimately limiting the increase in C_HLA_ [30]. This interlayer confinement mechanism was widely supported by previous clay mineral humification studies: the expandable 2:1 layered structure of Mont could accommodate small organic monomers but sterically hinder the formation of large-molecular-weight polycondensation products, resulting in smaller molecular size and lower polymerization degree of generated humus (HS) [31,40]. The FTIR results of Mont minerals (Section 3.4) further provided indirect evidence: the Si–O stretching peak intensity peaked at 0.06 mol/L Gly, indicating that Gly intercalation expanded the interlayer spacing and altered silicate framework vibration, which confirmed the occurrence of organic-mineral interactions within the interlayer domain. Conversely, Kao continuously activated reaction intermediates via abundant surface active sites without spatial restriction and constructed a local high-concentration microenvironment to enhance polymerization efficiency, resulting in a far greater C_HLA_ increment than the Mont system [40,41]. Benefiting from efficient surface catalysis and weak non-specific adsorption [42], the Kao system maintained significantly higher C_HLA_ levels throughout the reaction.

Gly addition exhibited a typical low-concentration promotion and high-concentration inhibition effect on humic C accumulation in both minerals, and 0.06 mol/L was identified as the optimal dosage [33]. The identification of 0.06 mol/L as the optimal Gly dosage was supported by both pre-experimental wide-range screening and multi-indicator cross-validation in the formal experiment. Although the formal experiment set five concentration gradients, the pre-experiment covered a wider range up to 0.48 mol/L and confirmed the unimodal dose-response relationship, ensuring that the observed optimum was not an artifact of limited concentration settings. At this concentration, Gly molecules bind adaptively to mineral active sites and provide sufficient amino donors for the Maillard reaction, which optimizes reaction efficiency and guarantees the continuous supply of intermediates [43]. Excess Gly at high dosages competitively occupied surface active sites and induced steric hindrance, which hindered substrate binding and intermolecular condensation. This inhibitory effect further reduced the polymerization degree of HLS and suppressed C_HLA_ accumulation [26].

Most reactants remained free in the solution microenvironment, which guaranteed the continuous transformation from FLA to HLA [44]. For the Mont system, strong interlayer adsorption and immobilization reduced the concentration of free reactive substrates, slowed down the FLA-to-HLA conversion rate, and led to a slow increase in the C_HLA_/C_FLA_ ratio [44]. After 240 h, the adsorption sites of Mont gradually saturated, and the reaction transformation process reached a relative balance, thus forming a distinct plateau stage of the C_HLA_/C_FLA_ ratio.

Differences in mineral structural characteristics also determine the aromaticity and structural complexity of humic products. Kao effectively promoted intermolecular condensation reactions, which are essential for the formation of highly aromatic HLS [45]. In contrast, the interlayer steric hindrance of Mont inhibited the polycondensation of aromatic compounds, resulting in HLS with lower aromatic condensation and simpler structures [46]. The dynamic variation of the E_4_/E_6_ ratio was consistent with the molecular evolution of HLS. The rapid proliferation of low-aromatic small molecular intermediates in the early stage led to an elevation of the E_4_/E_6_ ratio, with the peak value occurring at 76 h. After this critical time node, the consumption of initial substrates slowed down the generation of new intermediates, and low-molecular-weight fractions were gradually transformed into high-aromatic macromolecular HLS [47,48]. This metabolic transformation ultimately caused a progressive decline in the E_4_/E_6_ ratio in both mineral systems.

### 4.3. Effect of Gly concentration on atomic molar ratios of HLA, and FTIR spectra of HLA

With respect to the elemental molar ratios, the H/C ratio of Mont-mediated HLA was consistently higher than that in the Kao group. The interlayer spatial confinement of Mont restricted the cross-linking and aromatic condensation of aliphatic intermediates, thereby retaining more aliphatic functional groups and elevating the H/C ratio [14,40]. In contrast, the unconstrained surface reaction environment of Kao accelerated the aromatization of intermediates and reduced aliphatic components, resulting in lower H/C values [39,46]. Combined with the consistently lower E_4_/E_6_ ratio of Kao-derived HLA and the characteristic aromatic C = C absorption signal in FTIR spectra, multiple independent lines of evidence consistently supported that Kao promoted higher aromatic condensation and molecular maturity of HLS.

The C/N ratio in both systems showed an overall downward trend with increasing Gly concentration, as Gly acted as the core N donor to continuously incorporate N into HLA structures via C–N bond formation [13,18]. Mont’s interlayer adsorption stabilized N-containing intermediates and prevented N loss, leading to a steady decline in the C/N ratio [40]; Kao observed a transient fluctuation at 0.06 mol/L Gly due to the temporary mismatch between N supply and surface catalytic efficiency, but Gly-driven N enrichment still dominated the overall trend [9,39].

Both mineral groups presented a unimodal O/C ratio pattern peaking at 0.03 mol/L Gly. A low Gly dosage activated the Maillard reaction and synergistically promoted the generation of O-containing functional groups with clay catalytic sites, thereby increasing the O/C ratio [8,18]; excessive Gly competitively occupied catalytic active sites and inhibited oxidation, while enhanced polycondensation consumed partial O-containing functional groups, thus decreasing the ratio [26]. The consistent O/C variation trend indicated that Gly dosage exerted a stronger effect on the oxidation degree of HLA than mineral type, and clay structures regulated the formation and consumption of O-containing functional groups by affecting Maillard reaction kinetics. Notably, the maximum O/C ratio of the Kao-treated group (1.34) was higher than that of the Mont-treated group (1.02) at the optimal Gly concentration of 0.03 mol/L. This difference could be attributed to the stronger surface catalytic activity of Kao compared to Mont: under moderate Gly dosage, Kao’s surface active sites more effectively promoted the oxidation of HLA intermediates and the formation of O-containing functional groups [49], while Mont’s interlayer confinement slightly restricted the overgeneration of O-containing functional groups, resulting in a lower peak O/C ratio.

With respect to FTIR spectral characteristics, Mont-mediated HLA displayed stronger peak intensities at 3419–3443 cm^-1^ (–OH stretching) and 1621–1639 cm^-1^ (aromatic C = C stretching), which was attributed to interlayer confinement that preserved hydroxyl and aromatic fragments and hindered deep polycondensation [19]. Conversely, Kao-mediated HLA showed a higher peak intensity at 1384–1402 cm^-1^ (methyl C–H symmetric bending), since its surface catalysis promoted methylation and aliphatic methyl enrichment [32]. The polysaccharide C–O stretching peak at 1081–1103 cm^-1^ fluctuated much more widely in the Kao system, as its heterogeneous surface sites were highly sensitive to Gly gradients, while Mont’s interlayer stabilization weakened the fluctuation of this peak [14,32]. This significant difference in fluctuation range was closely related to the heterogeneous surface properties of Kao, which made it highly sensitive to changes in Gly concentration; in contrast, Mont’s stable interlayer environment buffered the impact of the Gly gradient on polysaccharide-related functional groups, thus minimizing the fluctuation of the C–O stretching peak intensity. The quantitative peak deconvolution results provide more accurate evidence for the structural differentiation of HLA mediated by the two clay minerals. The higher relative proportion of aromatic C = C groups in Kao-derived HLA directly verified its stronger ability to promote aromatic condensation, which was consistent with the E_4_/E_6_ and elemental analysis results.

### 4.4. Effect of Gly concentration on FTIR spectra of clay minerals

The broad absorption band at 3419–3443 cm^-1^ corresponds to the stretching vibration of intrinsic lattice –OH groups, a diagnostic signature for the crystal structure of clay minerals [21]. For Kao, the intensity of this –OH peak first increased and then decreased with rising Gly dosage, reaching a maximum (6.94) at 0.12 mol/L Gly. This trend arose from dynamic hydrogen-bonding interactions between Gly’s amino/carboxyl groups and Kao’s surface –OH sites: low-to-moderate Gly concentrations strengthened intermolecular hydrogen bonding and enhanced the –OH vibrational signal, while excessive Gly induced steric hindrance that disrupted these hydrogen bonds and weakened peak intensity [50]. In contrast, Mont exhibited significantly higher –OH peak intensities than Kao for all Gly treatments, which is attributed to its 2:1 layered structure with abundant interlayer lattice –OH groups and hydrated exchangeable cations that stabilize hydroxyl vibrational modes [5].

The absorption peak at 1621–1639 cm^-1^ is assigned to the bending vibration of H–O–H bonds in physically adsorbed water molecules on mineral surfaces and in interlayer domains [22]. Kao and Mont displayed opposite dynamic trends for this peak: the Kao H–O–H peak peaked at 0.06 mol/L Gly (intensity 0.43), as moderate Gly loading promoted the formation of secondary hydration shells around Gly-mineral complexes and enriched surface-adsorbed water [41]. Conversely, the Mont H–O–H peak reached a minimum (1.06) at 0.06 mol/L Gly, reflecting Gly-driven interlayer water displacement—Gly molecules intercalated into Mont’s expandable interlayer space, replacing physically adsorbed water and reducing the H–O–H vibrational signal [51]. The consistently higher H–O–H peak intensity in Mont further confirmed its stronger water adsorption capacity and larger interlayer water storage volume relative to Kao [52].

The strong absorption peak at 1081–1103 cm^-1^ represents the asymmetric stretching vibration of Si–O bonds in the SiO_4_ tetrahedron, the core structural unit of the clay mineral silicate framework [23]. The two minerals showed contrasting Si–O peak responses to Gly gradients: the Kao Si–O peak decreased to a minimum (61.02) at 0.06 mol/L Gly, indicating that Gly binding to Kao’s siloxane surface distorted local SiO_4_ framework symmetry and attenuated vibrational activity [53]. For Mont, the Si–O peak increased to a maximum (77.72) at 0.06 mol/L Gly, as Gly intercalation expanded Mont’s interlayer spacing, relieved structural stress, and enhanced the rigidity and vibrational intensity of the silicate framework [54]. This concentration was consistent with the optimal Gly dosage for C_HLA_ accumulation in both systems, confirming that 0.06 mol/L Gly triggered the strongest mineral-organic coupling for both clay minerals. These FTIR-detected changes in mineral surface functional groups and framework vibration provided indirect but solid evidence for mineral–organic interfacial interactions. The consistent response pattern of mineral surface properties and HLA accumulation further supported the core mediating role of mineral-organic coupling in abiotic humification. Consistent with the FTIR evidence of mineral-organic coupling, MAOC content showed a unimodal pattern with increasing Gly concentration, reaching the maximum at 0.06 mol/L for both minerals: 2.18 g/kg for Kao and 3.45 g/kg for Mont. Mont accumulated higher MAOC content with a larger proportion of stably bound fractions, which was attributed to the intercalation of organic molecules into the expandable interlayer space, where they were physically protected from desorption and degradation [26]. In contrast, Kao mainly adsorbed organic matter on external surfaces through weak interactions, resulting in lower MAOC content and weaker C stabilization capacity [14,16]. These findings directly explain why Mont maintains more stable humification kinetics despite lower HLA yield, as a large portion of humified products are sequestered in the mineral interlayer as stable MAOC.

### 4.5. PLS-SEM and mechanism analysis

Although Gly addition strongly stimulated HLS formation, these humified intermediates failed to independently support long-term C stabilization in the mineral-mediated Maillard reaction system. Mineral–organic coupling acted as a core mediator, governing the transformation of labile organic fractions into stable C forms. Mineral surfaces provided physical protection and reactive interfaces, facilitating organic retention and continuous transformation. Such regulatory characteristics are consistent with previous reports, in which clay minerals stabilized mineral-associated organic C through adsorption and complexation [13].

HLS molecular structure exerted a strong positive effect on structural complexity, indicating that improved aromaticity and condensation degree were the core drivers of HLS maturation and C stability. C sequestration showed no significant direct effect on structural complexity, demonstrating that HLS molecular traits, rather than C accumulation, determined humified structural properties. Stable C pools could provide a steady microenvironment to facilitate continuous HLS molecular modification, reflecting a subtle synergistic relationship between C preservation and structural evolution.

The transition from HLS accumulation to molecular structural variation reflected the progressive nature of abiotic humification. The critical contribution of HLS molecular structure to C sequestration confirmed that highly condensed humic matrices possessed stronger recalcitrance, which favored long-term terrestrial C storage. The linkage between C sequestration and HLS maturation illustrated that C stabilization was not a unidirectional process. Stabilized C fractions further promoted HLS molecular reorganization and structural optimization during humification [55]. Collectively, long-term C sequestration relied on the combined effect of mineral physical protection and inherent HLS structural evolution, rather than a single independent pathway [56]. The mineral-associated organic C pool serves as a critical link between mineral-organic coupling and long-term C sequestration. PLS-SEM pathway analysis combined with MAOC quantification confirmed that mineral-organic coupling not only regulated HLS structural evolution in the liquid phase, but also directly determined the size and stability of the mineral-bound C pool.

Compared with previous single-mineral or metal oxide-mediated Maillard humification research, this work provided the systematic molecular-level comparison between independent Mont and Kao reaction systems under five glycine concentration gradients. Distinct from static endpoint detection in Miura et al. [14] and fixed-Gly experiments in Fukuchi et al. [13], our dynamic sampling from 0–360 h combined Logistic5 and Boltzmann kinetic models quantitatively distinguished the stable humification transition characteristics of Mont and the strongly concentration-sensitive reaction response of Kao. Different from metal oxide catalysts dominated by redox catalysis, Mont regulated humification via interlayer steric confinement to restrict macromolecular crosslinking, while Kao relied on abundant external surface active sites to accelerate aromatic polycondensation—this crystal-structure-dependent dual regulatory mode had not been explicitly separated and verified in prior clay humification research.

From an application perspective, the findings of this study also provide mechanistic guidance for the controlled production of artificial HS. Recent advances have developed diverse artificial HS from different bioresources for agricultural and environmental applications. Marzban et al. [57] achieved selective humification via low-severity alkaline hydrothermal treatment, producing artificial peat with physicochemical properties highly comparable to natural peat while preserving the fibrous biomass framework; this work established a scalable continuous manufacturing route, representing the “structure-preserving” alkaline humification paradigm. Volikov et al. [58] utilized hydrothermally synthesized artificial HS as chelating carriers to fabricate bio-based manganese microfertilizers, verifying the metal-binding capacity and nutrient-synergizing value of artificial humic matter and expanding their functional application scenarios. Compared with the humification processes described above, the clay-mediated mild Maillard reaction system could produce HLS with tunable molecular structures and adjustable aromaticity. The optimal Gly dosage and mineral type screening revealed in this work can be applied to regulate the structural properties of artificial humic products, thus supporting the development of fit-for-purpose humic-based materials for soil C sequestration, controlled nutrient release, and contaminated soil remediation.

### 4.6. Study limitations and future perspectives

This study systematically revealed the differential mediation effects of Mont and Kao on Gly-driven abiotic humification, but several limitations should be acknowledged. First, direct mineral physicochemical characterization, including BET specific surface area, pore structure distribution, X-ray diffraction (XRD), particle size distribution, and zeta potential, was not performed. These analyses would provide more direct quantitative evidence for the interlayer spatial confinement mechanism of Mont and the differences in surface properties between the two clay minerals. Second, advanced molecular characterization techniques such as solid-state ^13^C nuclear magnetic resonance (^13^C NMR) and Fourier transform ion cyclotron resonance mass spectrometry (FT-ICR-MS) were not applied; these techniques can directly resolve the molecular composition and aromatic structure of HLS at the atomic level and will be included in follow-up research. Third, direct evidence for mineral–organic interfacial interactions can be further strengthened by X-ray photoelectron spectroscopy (XPS) surface element valence analysis and batch adsorption/desorption experiments of reaction precursors (Gly, Glu, Cat) on clay minerals. Fourth, this study was conducted in a simplified sterile laboratory simulation system within 360 h, which could not fully replicate the complex biotic-abiotic interactions and long-term evolutionary processes of natural soil ecosystems. The conclusions regarding C stabilization and humification regulation are therefore preliminary, and further field-based long-term experiments are needed to validate their practical applicability.

Future work will address the above limitations through multi-method joint characterization and targeted controlled experiments to establish a more comprehensive and rigorous mechanism of clay-mediated abiotic humification. In addition, denser Gly concentration gradients will be set in follow-up studies to more precisely locate the optimal reaction dosage. Quantitative FTIR peak deconvolution combined with X-ray photoelectron spectroscopy will be further applied to resolve the fine speciation of C and N functional groups during humification, and systematic C mass balance and desorption experiments will be conducted under more soil-like conditions to improve the environmental relevance of the findings.

## 5. Conclusion

Over the 360 h laboratory incubation, elevated Gly concentration continuously boosted the supernatant E_4_/E_6_ ratio and TOC content, reflecting enhanced partitioning of highly aromatic HLS to mineral surfaces. Kao induced a larger relative increment in supernatant E_4_/E_6_, while Mont maintained more stable kinetic transition points. The optimal Gly concentration for HLA-C accumulation was 0.06 mol/L in both clay systems. Kao facilitated higher aromatic condensation and molecular maturity of HLS, whereas Mont likely restricted macromolecular polymerization through interlayer spatial confinement. FTIR spectra confirmed distinct responses of the two clay minerals to Gly gradients and strengthened mineral–organic coupling at the optimal dosage. PLS-SEM modeling indicated that Gly drove HLS formation, and mineral-organic coupling may act as a key node associated with structural evolution and mineral-associated C retention. These findings reveal the structure-dependent regulatory patterns of typical clay minerals in abiotic humification under simulated conditions, providing a preliminary mechanistic basis for optimizing precursor concentrations to promote soil C stabilization; further long-term and field-scale studies are required to verify their ecological relevance to terrestrial ecosystems.

## Supporting information

S1 Data Original data. (ZIP)

## Abbrevations

MRPs: Maillard reaction precursors
HLS: Humic-like substances
HS: Humus
HLA: Humic-like acid
FLA: Fulvic-like acid
TOC: Total organic carbon
Gly: Glycine
Glu: Glucose
Cat: Catechol
Mont: Montmorillonite
Kao: Kaolinite
TOC: Total organic carbon
C_HLA_: Carbon content of humic-like acid
C_FLA_: Carbon content of fulvic-like acid
E_4_/E_6_: Absorbance ratio at 465 nm / 665 nm
MAOC: Mineral-associated organic C
FTIR: Fourier transform infrared spectroscopy
PLS-SEM: Partial least squares structural equation modeling
AIC: Akaike information criterion
